# Kangai Injection Combined with Platinum-based Chemotherapy for the Treatment of Stage III/IV Non-Small Cell Lung Cancer: A Meta-analysis and Systematic Review of 35 Randomized Controlled Trials

**DOI:** 10.7150/jca.31928

**Published:** 2019-08-28

**Authors:** Hongxiao Li, Yuejin Ji, Shiping Zhang, Zishan Gao, Cheng Hu, Rilei Jiang, Meijuan Chen, Guochun Li, Xu Zhang

**Affiliations:** 1 School of Medicine and Life Science, Nanjing University of Chinese Medicine, Nanjing 210023, China; 2 Jiangsu Collaborative Innovation Center of Traditional Chinese Medicine (TCM) Prevention and Treatment of Tumor, Nanjing University of Chinese Medicine, Nanjing 210023, China; 3 The Affiliated Hospital of Nanjing University of Chinese Medicine, Nanjing, 210029, China; 4 Clinical Acupuncture and Moxibustion Department, Second School of Clinical Medicine, Nanjing University of Chinese Medicine, Nanjing 210023, China; 5 Department of epidemiology and biostatistics, Nanjing University of Chinese Medicine, Nanjing 210023, China

**Keywords:** Kangai injection, platinum-based chemotherapy, non-small cell lung cancer, Meta-analysis, systematic review

## Abstract

**Objective**: In an effort to inform evidence-based guidelines for clinical practice, we performed a meta-analysis to systematically evaluate the safety and efficacy of Kangai injection (KAI) plus platinum-based chemotherapy for stage III/IV non-small cell lung cancer (NSCLC).

**Methods**: Randomized controlled trials (RCTs) comparing KAI plus platinum-based chemotherapy (experimental group) to chemotherapy alone (control group) were electronically retrieved from the Cochrane Library, PubMed, EMbase, Web of Science, Chinese National Knowledge Infrastructure (CNKI), Chinese Biological Medicine (CBM) Database, Wanfang Database, and the VIP Database for Chinese Technical Periodicals. RCTs published from the date of inception to July 5, 2018, were included. All trials were assessed for methodological quality in accordance with the Cochrane Reviewer's Handbook for Systematic Reviews of Intervention. Meta-analysis was performed using RevMan5.3 Software and Comprehensive Meta-Analysis (CMA) 2.0.

**Results**: The final analysis included 35 RCTs involving 2,618 patients. Our meta-analysis revealed that KAI combined with platinum-based chemotherapy was associated with significantly greater objective response rate (ORR) (RR=1.36, 95% CI: 1.25-1.49, *P*<0.00001) and disease control rate (DCR) (RR=1.14, 95% CI: 1.09-1.18, *P*<0.00001), improvements in quality of life (QOL) (RR=1.75, 95% CI: 1.59-1.93, *P*<0.00001), and decreases in the incidence of gastrointestinal reactions (RR=0.64, 95% CI: 0.54-0.77, *P*<0.00001), leukocytopenia (RR=0.54, 95% CI: 0.46-0.63, *P*<0.00001) and thrombocytopenia (RR=0.52, 95% CI: 0.36-0.76, *P*=0.0007) when compared with chemotherapy alone. In addition, combined treatment was associated with greater regulation of tumor immune function, as indicated by increases in the proportion of NK, CD_3_^+^ , and CD_4_^+^ cells (MD=2.27, 95% CI: 1.18-3.36, *P*<0.0001; MD=12.86, 95% CI: 11.64-14.08, *P*<0.00001; and MD=5.48, 95% CI: 2.68-8.28, P=0.0001) and decreases in the percentage of CD_8_^+^ cells (MD= -2.37, 95% CI from -4.51 to -0.23, *P*=0.03).

**Conclusions**: From the available evidence, our results indicate that KAI plus platinum-based chemotherapy could be more effective in improving clinical efficacy, decreasing the incidence of adverse reactions and regulating the tumor immune function than chemotherapy alone in the treatment of stage III/IV NSCLC. Nevertheless, considering the limitations of the included studies, rigorous designed, high-quality, multicenter clinical trials are still need to further confirm the results.

## Introduction

Globally, lung cancer remains the primary causes of cancer-related deaths 1, 2. Non-small cell lung cancer (NSCLC) accounts for approximately 85% of cases. The 5-year survival rate for NSCLC is less than 15% 3. Platinum-based chemotherapy as a conventional therapy is frequently utilized for the nonsurgical treatment of NSCLC due to its significant efficacy in improving tumor response rates 4-7. However, the adverse effects of chemotherapy have been associated with poor quality of life, low survival rates, and immune dysfunction. Thus, the need for supplementary treatments that improve the clinical efficiency of treatment, enhance immune function, and minimize adverse reactions during platinum-based chemotherapy remains urgent.

Traditional Chinese medicine (TCM) has attained great popularity in the alternative and complementary treatment of advanced NSCLC 8. Recent studies have indicated that platinum-based chemotherapy combined with TCM significantly improves efficiency and reduces toxicity relative to chemotherapy alone, which plays an irreplaceable role in clinical practice 9-11. As a kind of Chinese patent medicine (CPM), Kangai injection (KAI) is widely used in the treatment of NSCLC. It is an intravenous fluid made from an extraction of three Chinese herbs (i.e., ginseng, astragalus, and matrine). Its China Food and Drug Administration number is Z20026868. Active compounds of KAI include astragalus polysaccharides, astragalosides, ginsenosides, ginseng polysaccharide and oxymatfine 12, and various studies have shown that KAI possesses multiply pharmacological effects: (1)inducing tumor cell apoptosis 13, 14; (2)inhibiting tumor cell proliferation, invasion and metastasis 15, 16; (3)increasing the sensitivity of chemotherapy drugs and reducing adverse events caused by chemotherapy 17-19; (4)improving the body's immune function 20-22. In the clinic, Fan et al. found that KAI has obtained good curative effect, could ultimately prolong the survival and improve the quality of life, and mainly used in the treatment of multi-cancers, including liver, lung, colorectal cancer, etc. 23. In terms of animal experiments, Zhou et al. found that herbal formula astragalus polysaccharide and polysaccharopeptide could show immunomodulatory effects and anti-tumor activity in mice with lung cancer 24. Meanwhile, the results of Xie et al. showed that Ginsenoside Rg3 could obviously inhibit the volume and weight of tumor in xenografts model, and the mechanism of Ginsenoside Rg3 anti-tumor effects may be related with inhibiting PI3K/Akt signaling pathways 25.

Currently, a variety of trials have reported that KAI combined with chemotherapy could play a synergistic and attenuation role in NSCLC 26, 27, but, unfortunately, the conclusion is still controversial in different studies 28, 29. Previous meta-analysis have demonstrated that the combination of KAI and platinum-based chemotherapy could increase the efficacy, improve the quality of life and reduce the adverse reactions of NSCLC 30, 31, but none of the two studies evaluated the efficacy of combined therapy in regulating immune function as immunosuppression is a widely recognized complication of various chemotherapy regimens. Moreover, there have emerged some new studies evaluating the efficacy of KAI combined with platinum-based chemotherapy for NSCLC. Therefore, there is a need to update the systematic review and meta-analysis, for helping further and precisely reveal the efficacy, safety, and immune-enhancing effects of KAI, which will be beneficial to the treatment of advanced non-small cell lung cancer and the popularization of KAI.

## Materials and Methods

The present meta-analysis and systematic review were conducted in accordance with the Preferred Reported Items for Systematic Review and Meta-analysis (PRISMA) guidelines. Our systematic review is registered in the International Prospective Register of Systematic Reviews (PROSPERO) database (registration number: CRD42018103626).

### Literature source and search strategy

We searched the following electronic databases up to July 5, 2018: the Cochrane Library, PubMed, EMbase, Web of Science, Chinese National Knowledge Infrastructure (CNKI), Chinese Biological Medicine Database (CBM), Wangfang Database, and the VIP Database for Chinese Technical Periodicals (VIP). Two independent investigators searched for eligible according to the following search strategy: (“Lung Cancer”[Mesh] OR “Non-small Cell Lung Cancer”[Mesh] OR “Non-small Cell Lung Cancer” OR “NSCLC”) AND (“Chemotherapy” OR “chemotherapy” OR “chemotherapeutics” OR “chemical therapy”) AND (“Kangai injection”). A detailed search strategy of PubMed was shown in supplementary materials. Clinical studies published in languages other than English or Chinese were excluded.

### Inclusion criteria

Our analysis included randomized controlled trials (RCTs) comparing a platinum-based chemotherapy group (control) with a chemotherapy plus KAI group (experimental), regardless of blinding or allocation concealment. Eligible studies included patients diagnosed with Stage III-IV NSCLC via pathological or cytological examination, with Karnofsky performance scores (KPS) ≥60 or survival times ≥3 months. Eligible patients were required to exhibit no obvious abnormalities in the liver, kidney, cardiac function, or contraindications for chemotherapy. Additional inclusion criteria were as follows: no age, gender, race, nationality, or regional restrictions in either arm; no significant differences in baseline conditions between the experimental and control groups (P > 0.05); inclusion of at least one outcome indicator (objective response rate, quality of life, adverse effects, immune function).

### Exclusion criteria

Studies involving patients with other primary tumors; those with severe cardiovascular, hepatic, or renal diseases; and those in which treatment was combined with surgery, radiotherapy, or other TCM therapies were excluded. Non-RCTs (animal experiments, case reports, cohort studies, review articles, etc.), duplicate studies, and studies reporting no outcome measures were also excluded. Studies in which KAI was administered non-intravenously (e.g., oral granules) and those of insufficient quality (i.e., incomplete information, obvious error, inappropriate statistical methods, non-rigorous experimental design, etc.) were excluded as well.

### Outcome indicators and evaluation criteria

The objective tumor response rate (ORR) and disease control rate (DCR) were evaluated in accordance with World Health Organization (WHO) evaluation criteria or the Response Evaluation Criteria in Solid Tumors (RECIST). Responses were categorized as complete relief (CR), partial remission (PR), stable disease (SD), or progressive disease (PD). ORR was calculated as follows: (CR + PR) / total number of cases × 100%, and DCR was calculated as follows: (CR + PR + SD) / total number of cases × 100%.

The KPS was used to assess the patient's quality of life (QOL). Patients exhibiting KPS increases or decreases of more than 10 points were considered to have experienced improvement or deterioration, respectively. QOL was considered stable in patients exhibiting changes within this range. Rates of improvement were calculated as follows: number of patients exhibiting improvement / total number of cases × 100%.

We adopted the WHO Recommendations for Grading of Acute and Subacute Toxicity to evaluate the adverse reactions of chemotherapy, which were classified into five levels: 0, I, II, III, and IV. In this meta-analysis, we examined only gastrointestinal reactions (nausea and vomiting) and myelosuppression (leukocyte, hemoglobin and platelet) of grade II or higher.

The incidence of alterations in cellular immune function (i.e., natural killer cells (NK), CD_3_^+^, CD_4_^+^, CD_8_^+^, and CD_4_^+^/CD_8_^+^ ratio) was also used to estimate the efficacy of KAI. analysis of immune alterations in included studies were performed using immunocytochemistry or flow cytometry.

### Data extraction and quality assessment

Two investigators (Hongxiao Li and Yuejin Ji) independently extracted and cross-checked the general characteristics of eligible studies. Discrepancies were resolved via discussion with a third reviewer (Shiping Zhang). The following features were extracted from each article: name of the first author, publication year, number of patients in each group, sex, age, physical status, tumor stage, intervention details (course of treatment, chemotherapy regimens, etc.), outcome indicators. For trials reporting incomplete or ambiguous data, we contacted the corresponding author via email for clarification. If no response was obtained after three attempts, the article in question was removed from the analysis. Two reviewers (Hongxiao Li and Yuejin Ji) evaluated the methodological quality of each included RCT based on the following items, in accordance with the Cochrane Collaboration's Risk of Bias criteria 32: random sequence generation (selection bias), allocation concealment (selection bias), blinding of participants and personnel (performance bias), blinding of outcome data (detection bias), incomplete outcome data (attrition bias), selective reporting (reporting bias), and other biases.

### Statistical analysis

As required by the Cochrane Collaboration, we utilized Review Manager 5.3 software and Comprehensive Meta-Analysis (CMA) 2.0 for the present meta-analysis. Risk ratios (RRs) and mean differences (MDs) were used to express therapeutic efficacy for dichotomous outcomes and continuous data, respectively. Both RRs and MDs were calculated using the effect value and 95% confidence intervals (CI). P values <0.05 were considered to indicate statistical significance. Inconsistency (*I^2^*) was used to detect the heterogeneity between studies, and a fixed-effects model was applied to calculate the pooled statistics when there was no significant heterogeneity (*I^2^*<50%). In other cases, a random-effects model was adopted (*I^2^*>50%). The stability of pooled results was confirmed by sensitivity analysis, which were performed using three methods: altering the combined-effects model, removing the study with the greatest weight, and exclusion of studies one-by-one. Moreover, funnel plots and Egger's tests were used to estimate the potential publication bias. Trial sequential analysis (TSA) was performed to evaluate the robustness of the results and meta-analytic sample size, while meta-regression analysis (MRA) was performed to explore the effects of potential heterogeneity and confounders on outcomes.

## Results

### Search results

**Figure 1:**
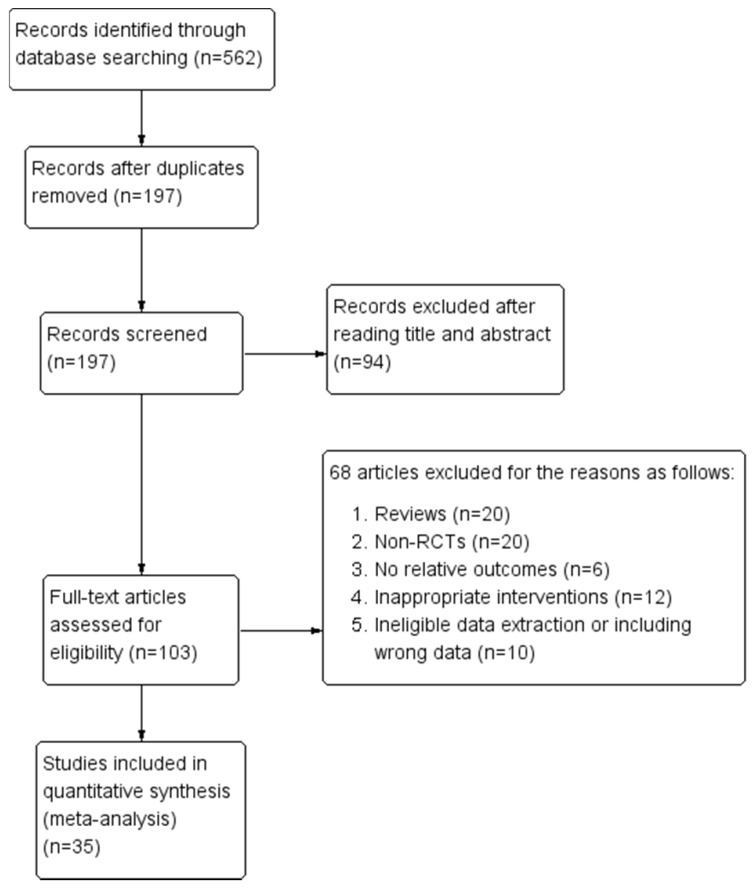
Flow diagram of screening process for eligible articles.

Our initial search identified a total of 562 relevant articles. Following the exclusion of 197 duplicate articles and a review of the remaining titles and abstracts, we evaluated the full text of 103 articles according to inclusion and exclusion criteria. An additional 68 articles were excluded mainly because they were reviews, non-RCTs, did not report relative outcomes, utilized inappropriate interventions or data extraction methods, and included data errors. In total, 35 RCTs were included in this meta-analysis. The screening process and results are shown in were shown in **Figure 1**.

### Basic characteristics of the included studies

A total of 2,618 patients were enrolled in the 35 included studies 26, 27, 33-65, all of which were conducted and published in China. Across these 35 studies, there were 1,328 and 1,290 patients in the experimental and control groups, respectively. Participant ages ranged from 21 to 85 years. Navelbine® (vinorelbine) plus platinum (NP) was the most frequently utilized chemotherapy regimen, followed by Taxol® (paclitaxel) plus platinum (TP), docetaxel plus platinum (DP), gemcitabine plus platinum (GP), 5-fluorouracil plus platinum (FP), pemetrexed plus platinum (PP), and vindesine plus platinum (VP). A total of 33 articles 27, 33-41, 43-65 reported ORR, 32 articles 27, 33-41, 43-53, 55-65 reported DCR. In addition, 27 studies 27, 36-38, 40-56, 59-62, 64, 65 assessed QOL using the KPS. Twelve studies 36, 37, 39, 41, 43, 44, 48, 52, 53, 55, 57, 62 provided outcomes regarding gastrointestinal reactions, while 15 provided outcomes regarding leukocytopenia 27, 36, 37, 39-41, 43, 44, 48, 52, 53, 55, 57, 62, 64, 4 documents reported hemoglobin deficiency 36, 37, 43, 64, and 6 studies assessed thrombocytopenia 36, 37, 43, 57, 62, 64. Immune function outcomes were assessed in nine trials 33-35, 42, 50-52, 61, 64. The basic characteristics of the included studies are shown in **Table 1**.

### Evaluation of methodological quality

**Figure 2:**
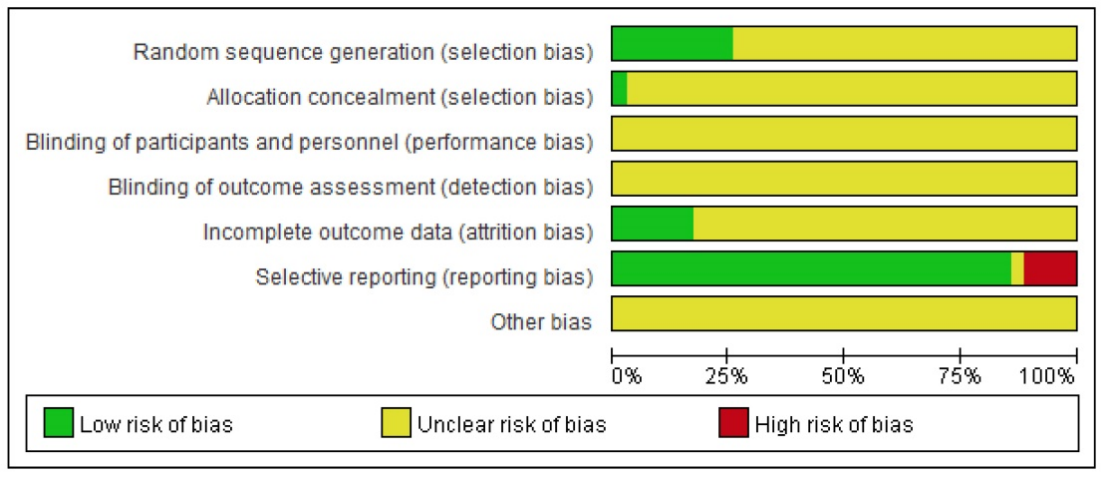
Summary of the risk of bias for each included study. “+” (green): low risk of bias; “?” (yellow): unclear risk of bias; “-” (red): high risk of bias.

**Figure 3:**
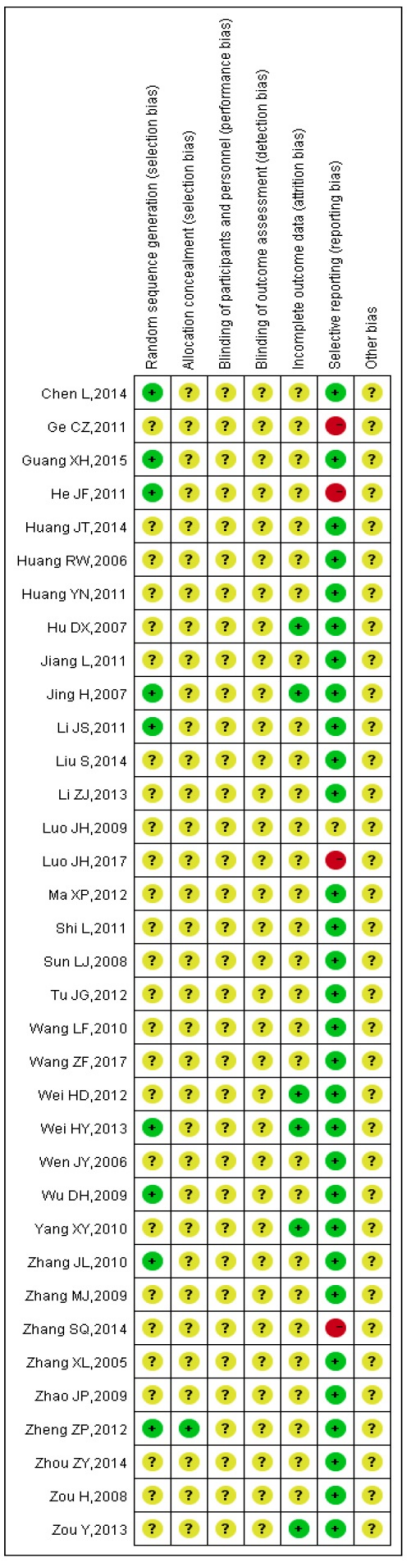
Risk of bias graph. Each risk of bias is presented as the percentage across all included studies.

The detailed results of the methodological evaluation are shown in **Figures 2**, **3** and Table S1. While all articles mentioned randomization, only nine trials indicated the specific method of random allocation 27, 34, 42, 47, 51, 53, 57, 63, 64. Only one study reported allocation concealment, which was implemented using an opaque envelope 42. None of the included studies described double-blinding or blinding of outcome assessments. Six studies reported rates of follow-up and withdrawal (attrition bias) 39, 44, 50, 57, 58, 64. With regard to reporting bias, because we observed that the results reported by four studies were less than the evaluation index 26, 38, 45, 63, these four studies were classified as “high risk”. Due to insufficient data among the retrieved studies, results for other forms of bias were unclear.

### ORR

**Figure 4:**
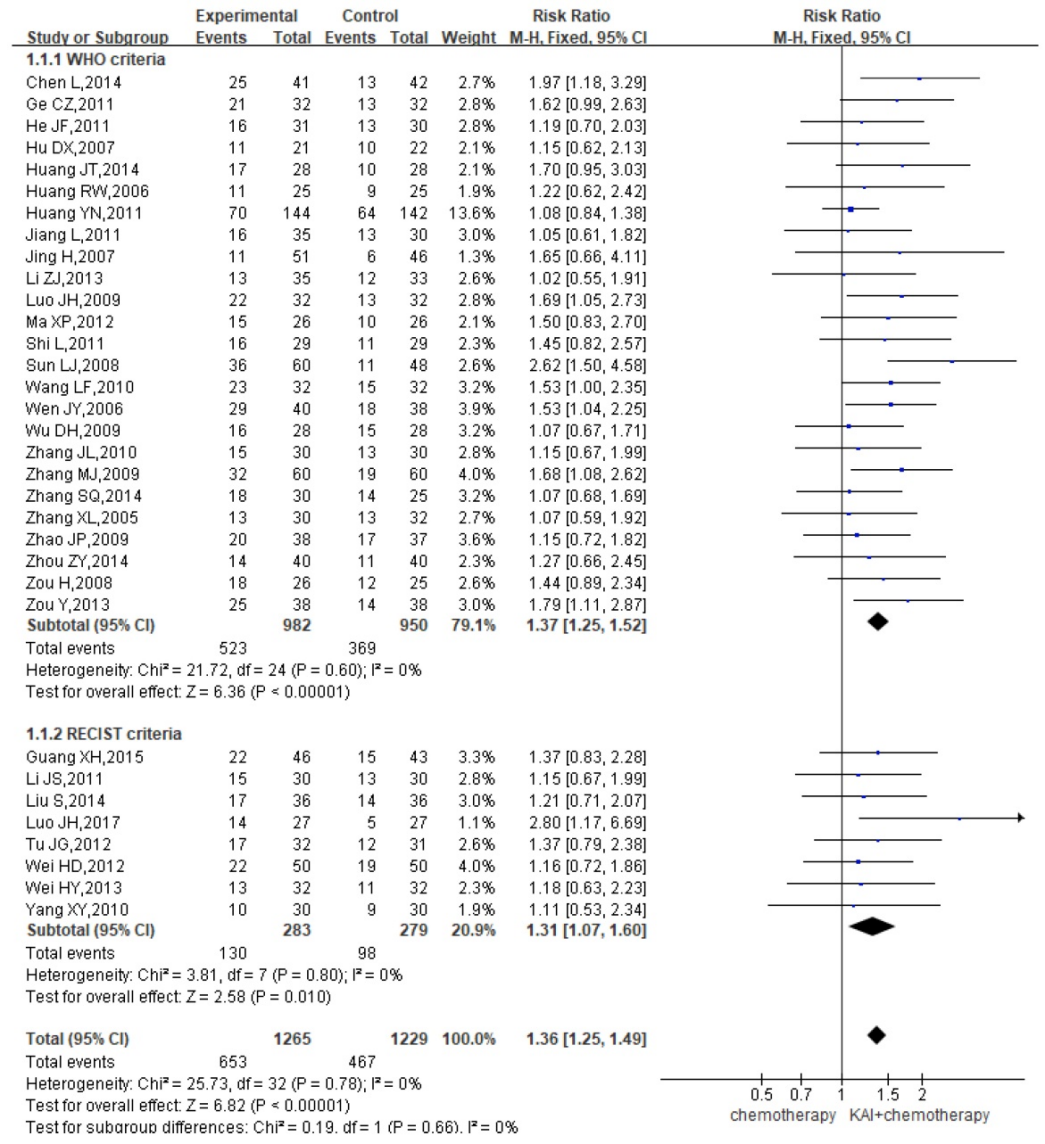
Forest plot showing objective response rates (ORR).

A total of 33 studies reported the ORR, yielding a total sample of 2,494 patients (1,265 in the experimental group and 1,229 in the control group). Subgroup analyses were performed based on whether WHO or RECIST criteria were utilized. Heterogeneity analysis revealed that all included studies exhibited good homogeneity with regard to the consistency of their results (*P*=0.78, *I^2^*=0%) (**Figure 4**). Therefore, the fixed-effects model was applied. Our results indicated that ORR were significantly greater among patients in the KAI group than among those receiving chemotherapy alone (WHO criteria: RR=1.37, 95% CI: 1.25-1.52, *P*<0.00001; RECIST criteria: RR=1.31, 95% CI: 1.07-1.60, *P*<0.010; overall effect: RR=1.36, 95% CI: 1.25-1.49, *P*<0.00001).

### DCR

**Figure 5:**
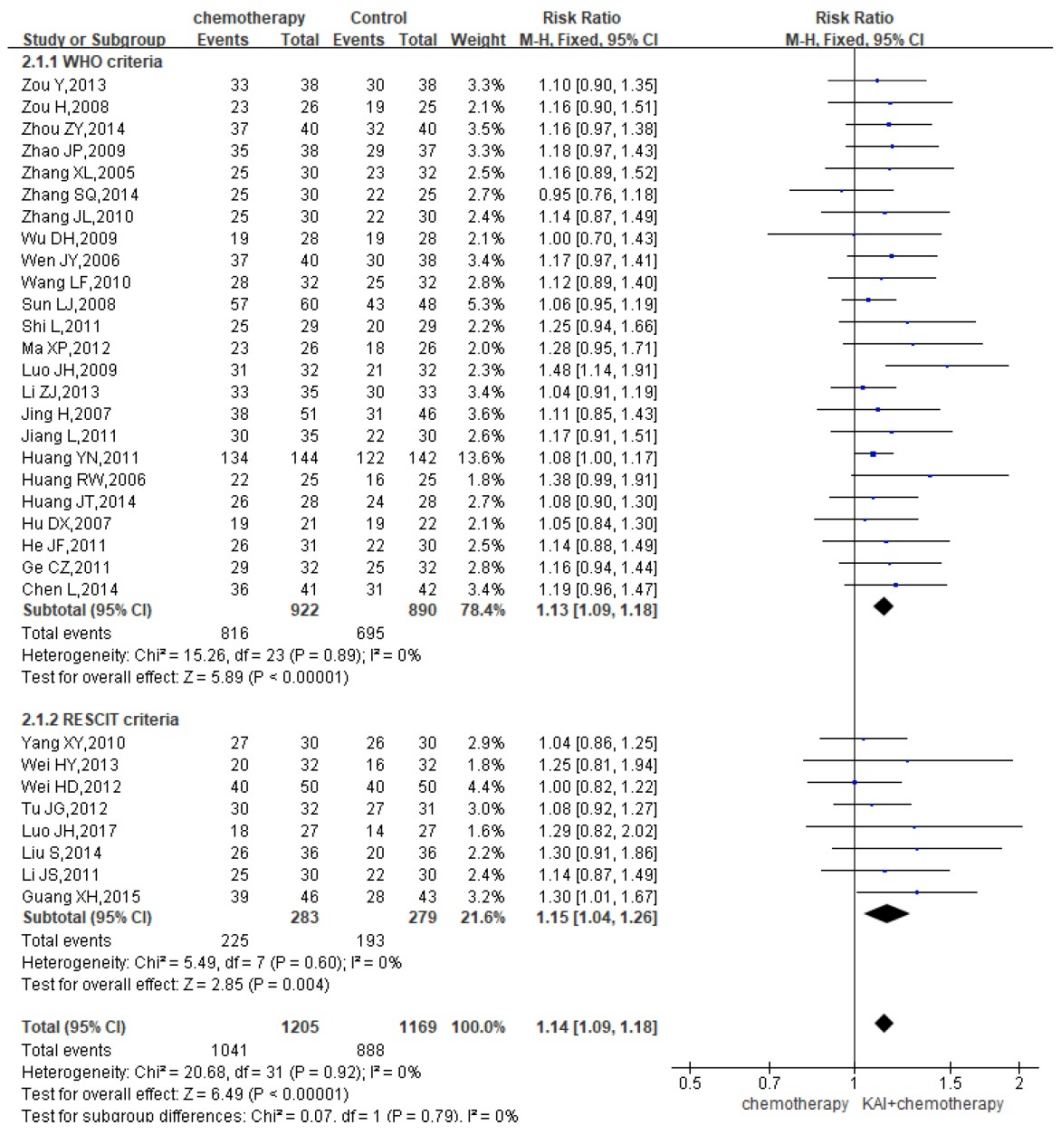
Forest plot showing disease control rates (DCR).

A total of 32 studies reported the DCR, yielding a total sample of 2,374 patients (1,205 in the experimental group and 1,169 in the control group). We performed subgroup analysis due to the different criteria. Heterogeneity analysis revealed that there is a good homogeneity with regard to the consistency of the results (*P*=0.92, *I^2^*=0%) (**Figure 5**). Therefore, the fixed-effects model was applied. Our results showed a significant improvement of DCR among patients in the KAI group (WHO criteria: RR=1.13, 95% CI: 1.09-1.18, *P*<0.00001; RECIST criteria: RR=1.15, 95% CI: 1.04-1.26, *P*=0.004; overall effect: RR=1.14, 95% CI: 1.09-1.18, *P*<0.00001).

### QOL

**Figure 6:**
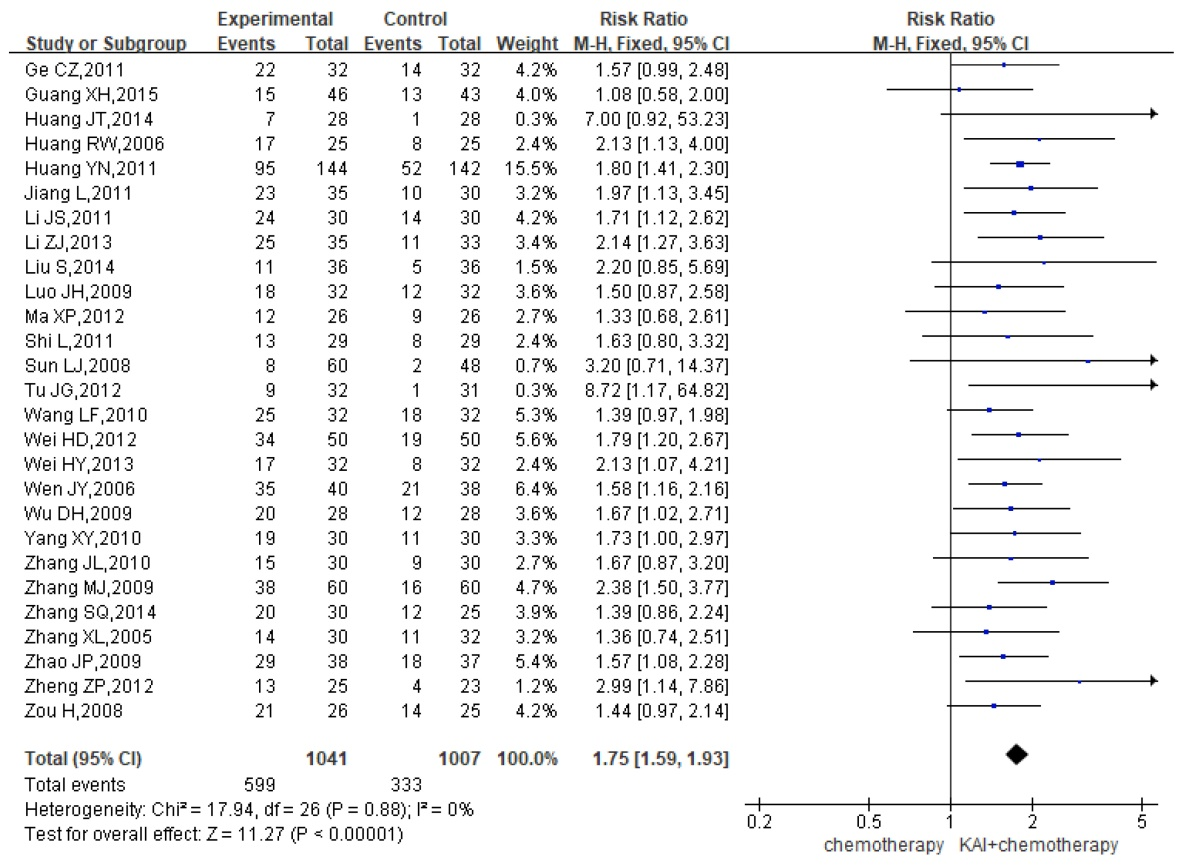
Forest plot showing quality of life (QOL) results for the included studies.

A total of 27 studies assessed QOL, yielding a total sample of 2,048 patients (1,041 in the experimental group and 1,007 in the control group). As shown in **Figure 6**, we utilized a fixed-effects model to assess the homogeneity of the included studies (*P*=0.88, *I^2^*=0%). Overall RRs suggested that the combination of KAI and chemotherapy was more effective at improving QOL than chemotherapy alone (RR=1.75, 95% CI: 1.59-1.93, *P*<0.00001).

### Gastrointestinal reactions

**Figure 7:**
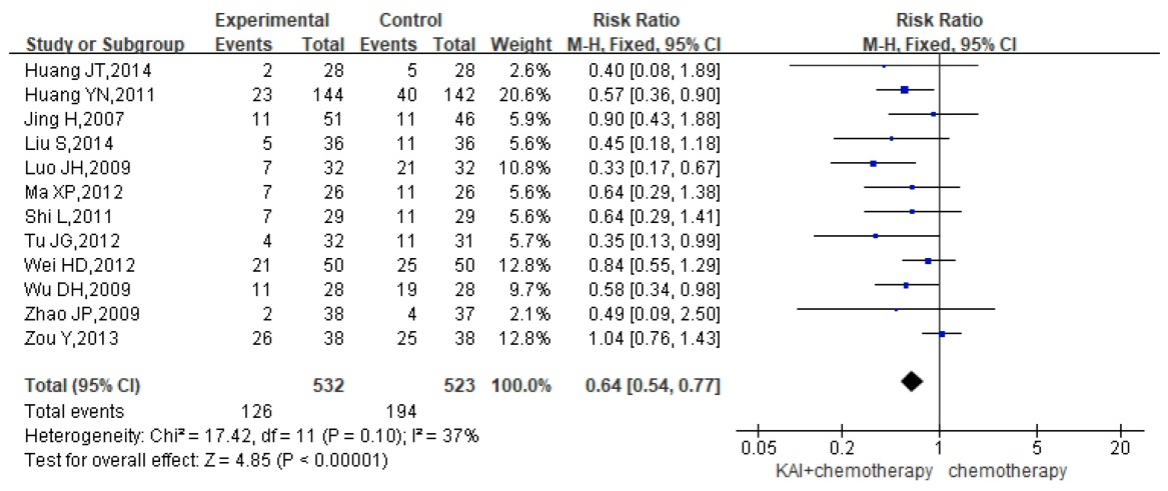
Forest plot showing the incidence of gastrointestinal reactions for the included studies.

Twelve studies reported the rate of gastrointestinal reactions, yielding a total sample of 1,055 patients (532 in the experimental group and 523 in the control group). As there was no significant heterogeneity in the results (*P*=0.10, *I^2^*=37%) (**Figure 7**), we performed quantitative data synthesis using a fixed-effects model. Overall RRs suggested that the combination of KAI and chemotherapy (RR=0.64, 95% CI: 0.54-0.77, *P*<0.00001) was more effective in reducing the incidence of gastrointestinal reactions than chemotherapy alone.

### Myelosuppression

**Figure 8:**
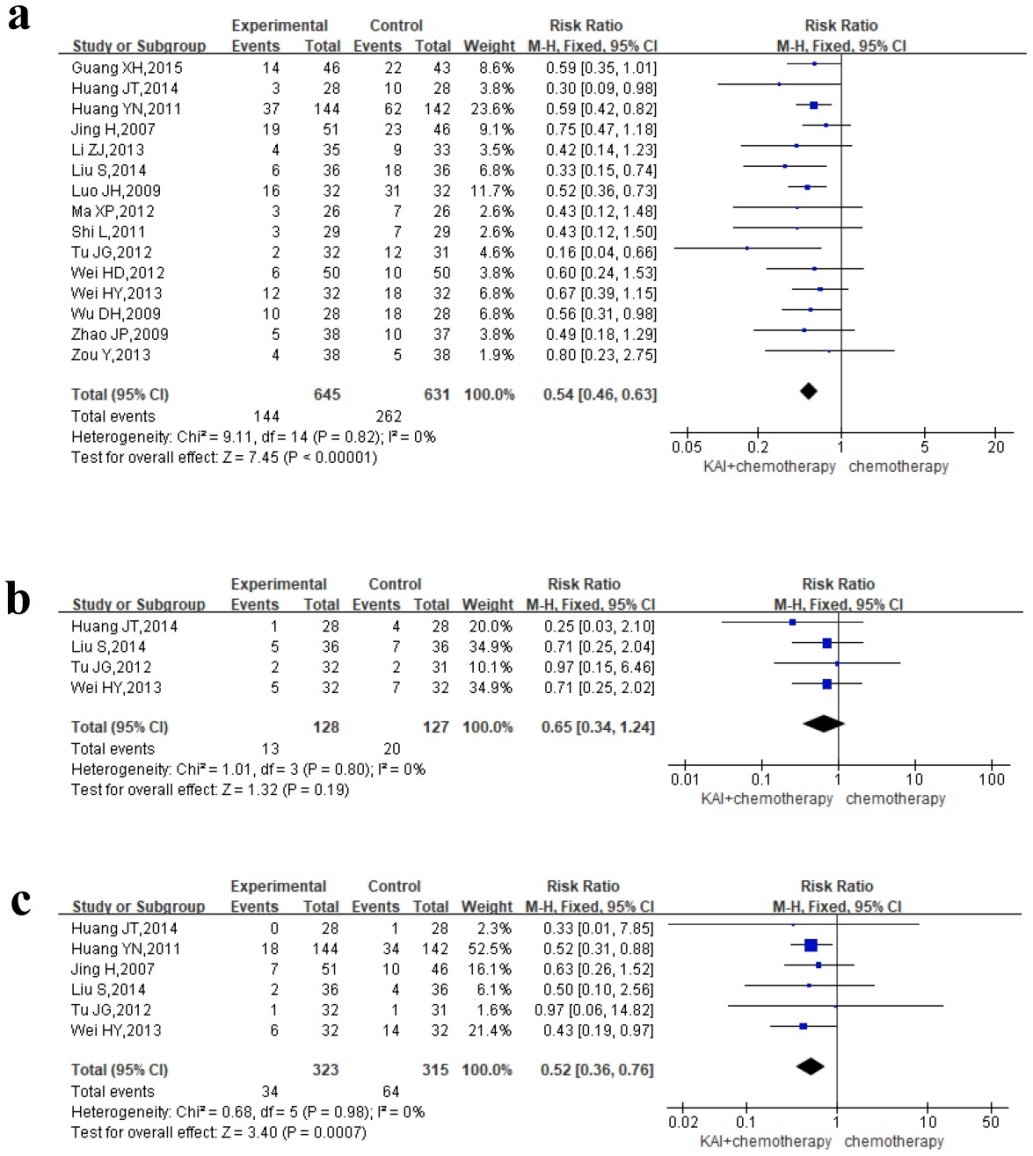
Forest plot showing rates of myelosuppression for the included studies: a. leukocytopenia; b. hemoglobin deficiency; c. thrombocytopenia.

To evaluate the role of KAI in improving myelosuppression rigorously, we performed meta-analysis of leukocyte, hemoglobin and platelet, respectively. Of all the studies included, 15 documents reported the incidence of leukocytopenia, 4 studies reported hemoglobin deficiency, and 6 studies assessed thrombocytopenia. We conducted a fixed-effects model for quantitative data synthesis according to the homogeneity of the results: leukocytopenia (*P*=0.82,* I^2^*=0%), hemoglobin deficiency (*P*=0.80,* I^2^*=0%), and thrombocytopenia (*P*=0.98,* I^2^*=0%) (**Figure 8a-c**). It is meaning for us to find that KAI combined with platinum-based chemotherapy could only reduce the incidence of leukocytopenia (RR=0.54, 95% CI: 0.46-0.63, *P*<0.00001) and thrombocytopenia (RR=0.65, 95% CI: 0.34-1.24, *P*=0.19) in this meta-analysis, while there is no significant difference in hemoglobin deficiency between the two groups (RR=0.52, 95% CI: 0.36-0.76, *P*=0.0007).

### Immune function

**Figure 9:**
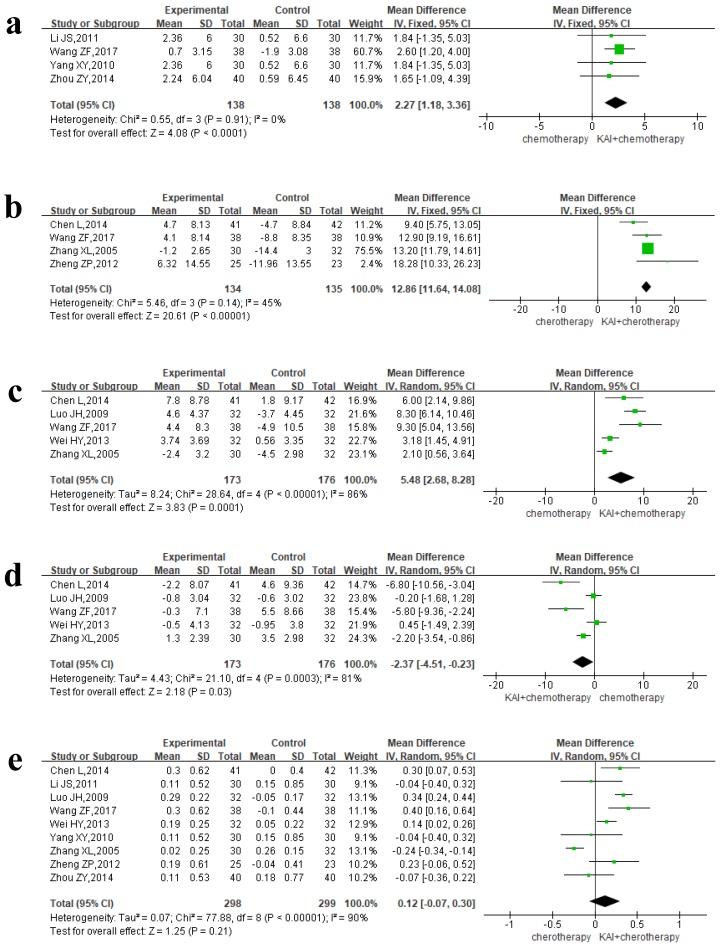
Forest plot shows immune index. a. NK; b. CD3^+^; c. CD4^+^; d. CD8^+^; e. CD4^+^/CD8^+^.

Four separate articles reported NK and CD_3_^+^ data, yielding 276 patients in the NK group and 269 patients in the CD_3_^+^ group. A fixed-effects model was used for quantitative data synthesis due to the homogeneity of the results: NK (*P*=0.91,* I^2^*=0%), CD_3_^+^ (*P*=0.14,* I^2^*=45%) (**Figure 9a-b**). Analysis of the pooled MD revealed that KAI combined with chemotherapy significantly improved NK (MD=2.27, 95% CI: 1.18-3.36, *P*<0.0001) and CD_3_^+^ values (MD=12.86, 95% CI: 11.64-14.08, *P*<0.00001) when compared with chemotherapy alone.

Due to the heterogeneity of CD_4_^+^ (P<0.00001,* I^2^*=86%), CD_8_^+^ (P=0.0003,* I^2^*=81%), and CD_4_^+^/CD_8_^+^ (P<0.00001,* I^2^*=90%) data (**Figure 9c-e**), we utilized a random-effects model for data synthesis. Our results indicated that the combination of KAI and chemotherapy significantly increased the percentage of CD_4_^+^ cells (MD=5.48, 95% CI: 2.68-8.28, *P*=0.0001) and significantly decreased the percentage of CD_8_^+^ cells (MD= -2.37, 95% CI from -4.51 to -0.23, *P*=0.03). However, there was no significance difference in CD_4_^+^/CD_8_^+^ ratio between the two groups (MD= 0.12, 95% CI from -0.07 to 0.30, *P*=0.21).

### Publication bias

**Figure 10:**
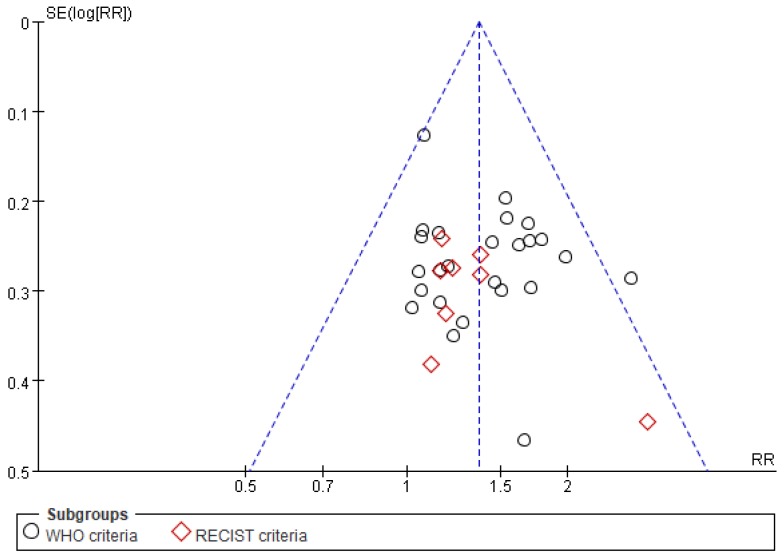
Funnel plot of ORR.

Funnel plots and Egger's tests were used to examine the potential publication bias among studies reporting ORR. As shown in **Figure 10**, although one point lies outside of the funnel, the plot is nearly symmetric, suggestive of a lack of publication bias. In addition, Egger's tests revealed that there was no significant bias with regard to ORR (*P*=0.285), consistent with the results of funnel plot analysis.

### Sensitivity analysis

Based on the results of our meta-analysis, we performed a sensitivity analysis for outcomes with high heterogeneity: CD_4_^+^, CD_8_^+^, and CD_4_^+^/CD_8_^+^. This analysis indicated that the results of the fixed-effects and random-effects models were consistent, suggesting that the results of our meta-analysis were stable and reliable. We then removed studies with the greatest weight and those exhibiting significant differences (**Table 2**), which reversed the resultant heterogeneity, suggesting that the excluded articles may have been the source of heterogeneity. Re-evaluation of the excluded studies revealed that differences in the dosage and course of chemotherapy may have been responsible for these results.

**Figure 11:**
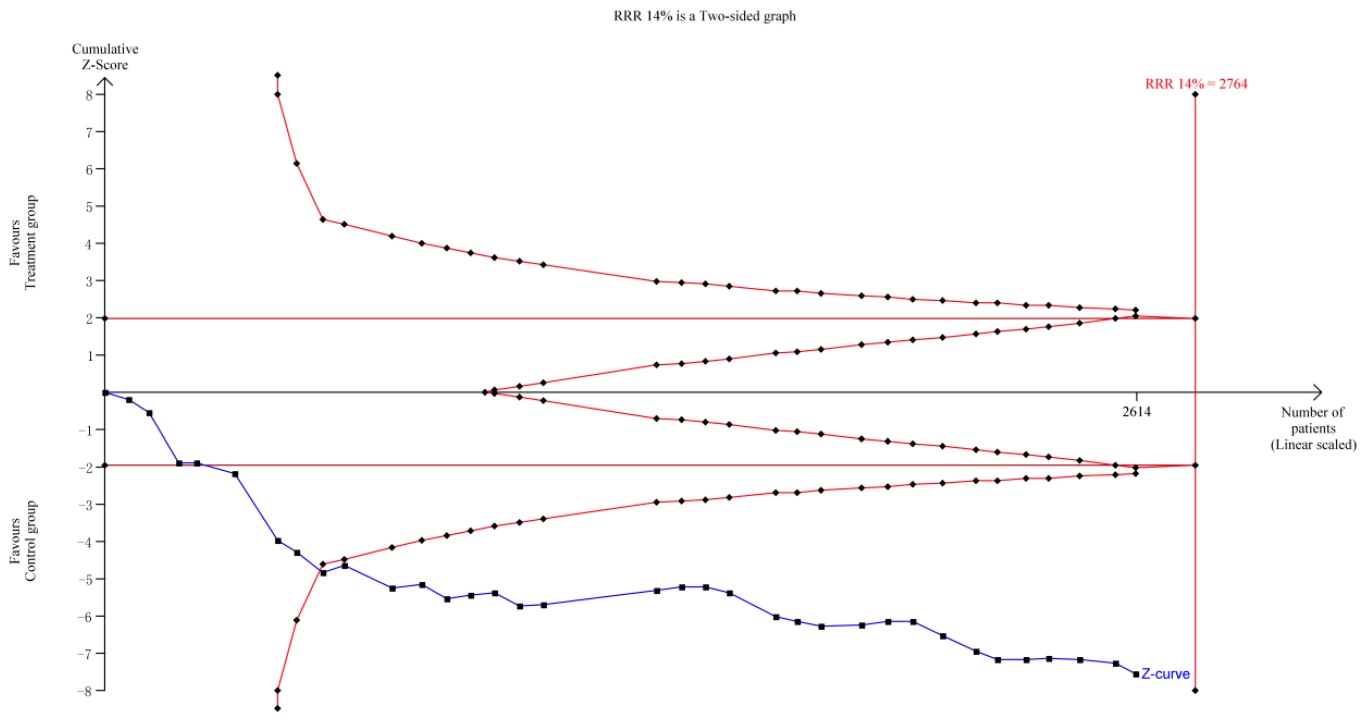
Trial sequential analysis on ORR.

TSA indicated that the required information size (RIS) for a reliable and conclusive meta-analysis had been reached, and that KAI plus chemotherapy was significantly superior to the control intervention (**Figure 11**). These findings suggest that overall results for the analysis of ORR were robust. However, the quality of some included studies was low, which may have influenced estimates of sample size. including some studies is low, it will produce significant influence for the estimation of sample size.

**Figure 12:**
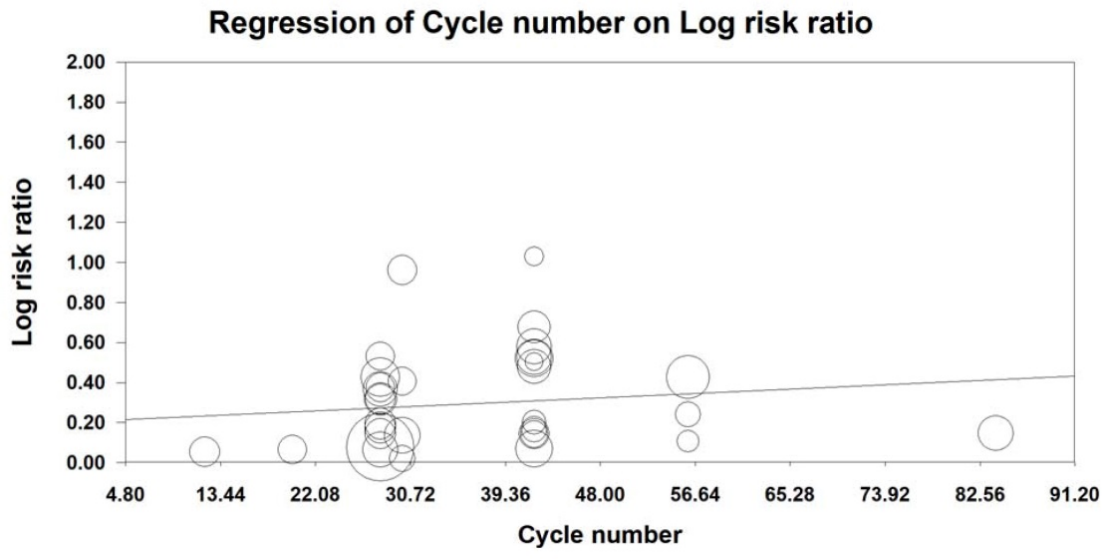
Meta-regression analysis on ORR.

**Figure 13:**
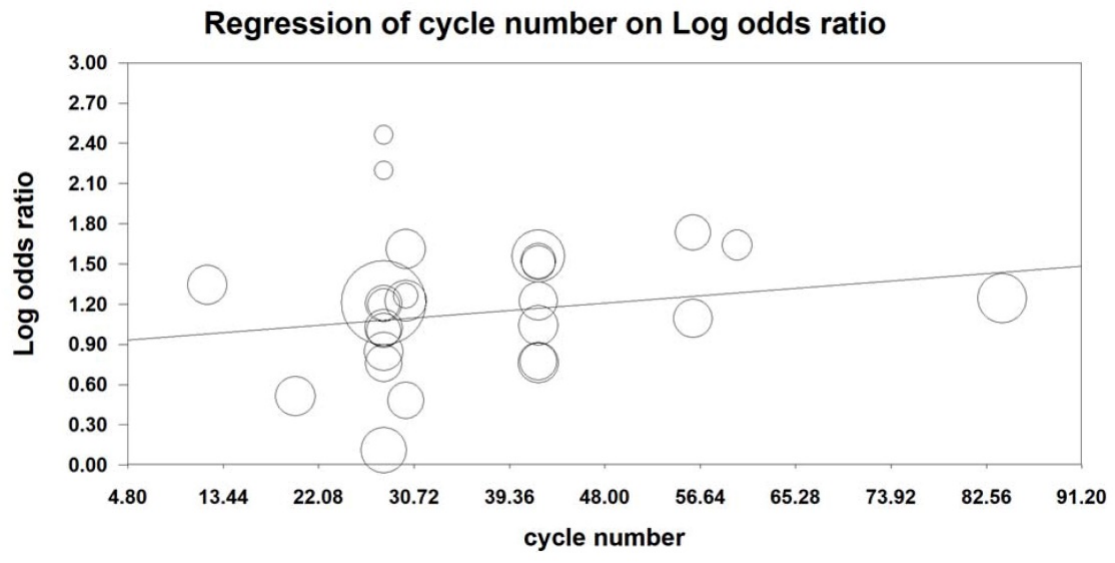
Meta-regression analysis on QOL.

MRA was also conducted to examine the effect of the number of KAI cycles on ORR and KPS. Our analysis revealed that KAI injection did not exert dose-dependent effects on ORR [logOR= -0.009-0.014 cycle number, (u=0.432, p=0.666)] or QOL [logOR=-0.006-0.019 cycle number, (u=0.987, p=0.324)]. Furthermore, both of these indices seemed to increase as the number of KAI cycles increased (**Figures 12, 13**).

## Discussion

### Efficacy and safety analysis

NSCLC is a common respiratory malignancy in China. Although platinum-based chemotherapy has improved the clinical efficiency of NSCLC treatment, the adverse reactions and immunosuppression associated with treatment significantly impact patient QOL, which may lead to discontinuation of treatment. Previous studies have demonstrated that KAI replenishes qi and enhances immune function when utilized as an adjuvant treatment for chemotherapy. Such findings have been observed among patients with primary liver cancer, colorectal cancer, and patients with NSCLC 66, 67. Modern pharmacological studies have reported that ginseng can inhibit tumor cell growth and differentiation, increase the sensitivity of chemotherapy drugs, and enhance the immune function of peripheral blood lymphocytes in patients with cancer 68. Astragalus has been reported to promote the induction of interferon or exert interferon-like effects, and to enhance anti-tumor effects by strengthening the activity of NK cells 69, 70. Additional research has indicated that matrine inhibits the proliferation and metastasis of tumor cells by inducing apoptosis, halting the cell cycle, and inhibiting the formation of blood vessels 71. Hence, we conducted a meta-analysis to evaluate clinical efficiency, adverse reactions, and immune function in patients treated with platinum-based chemotherapy plus KAI for stage III/IV NSCLC.

Our systematic review included 35 studies involving a total of 2,618 patients. TSA of ORR suggested that the RIS for a conclusive and reliable meta-analysis had been reached, and that the combination of KAI and chemotherapy was significantly more effective than chemotherapy alone.

ORR, DCR and QOL play an important role in assessing the clinical efficiency of NSCLC treatment. In this meta-analysis, we performed the subgroup analysis of ORR and DCR determined based on different criteria. This analysis revealed that KAI in combination with chemotherapy appeared to be more effective in improving ORR and DCR regardless of the criteria adopted. Our results further demonstrated that adjuvant treatment with KAI was associated with significant improvements in QOL as measured via KPS, suggesting that combined treatment enhances the tolerance to chemotherapy.

Although platinum-based chemotherapy can improve the efficiency of tumor treatment and QOL, the adverse reactions caused by chemotherapy may lead to poor rates of treatment adherence among patients. Indeed, chemotherapy frequently induces gastrointestinal reactions and myelosuppression. In order to evaluate the condition of myelosuppression rigorously, we performed meta-analysis of leukocyte, hemoglobin and platelet, respectively. Our meta-analysis revealed that adjuvant therapy with KAI may attenuate gastrointestinal reactions, reduce the incidence of leukocytopenia and thrombocytopenia of grade II or higher, while there was no significant difference in hemoglobin deficiency between the two groups.

The present meta-analysis marks the first attempt evaluate the effect of KAI on immune function during chemotherapy. Our results indicated that KAI combined with chemotherapy was significantly more effective in increasing the percentage of NK, CD_3_^+^, and CD_4_^+^ cells, and in decreasing the percentage of CD_8_^+^ cells, when compared with chemotherapy alone. Furthermore, there was no significant difference in CD_4_^+^/CD_8_^+^ between the two groups. Taken together, these results suggest that KAI exert beneficial effects on chemotherapy-induced immune dysfunction. We then performed sensitivity analysis due to the high heterogeneity for CD_4_^+^, CD_8_^+^, and CD_4_^+^/CD_8_^+^ indices. Although our results indicated good consistency among results, the observed heterogeneity may have influenced the stability and feasibility of our results.

Due to differences in tolerability and reactivity, KAI and chemotherapy regimens vary across patients. Therefore, we performed MRA of ORR and QOL, which revealed that KAI did not exert dose-dependent effects on ORR and QOL. Moreover, both indices seemed to increase as the number of KAI cycles increased.

### Limitations of the included studies

Although our results indicated that KAI combined with platinum-based chemotherapy is superior to chemotherapy alone with regard to improving clinical efficiency, reducing adverse reactions, and enhancing immune function, the included studies possess several limitations of note. First, although random allocation was utilized in all included studies, only nine of these studies reported the specific method of random allocation. While one study mentioned that opaque envelopes were used for allocation concealment, no such information was reported in the remaining studies, which may have resulted in selection bias. In addition, none of the 35 studies described the method of double-blinding or the blinding of outcome assessments. Only six articles reported rates of follow-up and withdrawal. Moreover, the results reported by four studies were less than the evaluation index in previous studies, suggestive of a certain degree of reporting bias. As the data were insufficient for the retrieved studies, sources of other bias remained unclear. In general, these potential sources of bias may have led to overestimation of curative efficiency, attenuating the strength of our conclusions. Second, it is possible that false-positive results were included, as the studies examined utilized an “A+B” to “B” design without strict placebo controls. Third, because all included studies were published in Chinese, language bias may have influenced our results. Given these concerns, our results should be interpreted with caution and verified in more rigorous trials with high methodological quality.

## Conclusions

Our findings suggest that KAI combined with platinum-based chemotherapy is effective in improving ORR, DCR and QOL, attenuating chemotherapy-induced adverse effects, and regulating immune function in patients with NSCLC. Thus, KAI may be appropriate for use as a complementary and alternative treatment for NSCLC. However, given the poor methodological quality of the included studies, our results should be interpreted with caution. Future RCTs with more rigorous designs should be conducted to yield more reliable conclusions to inform clinical practice.

## Supplementary Material

Supplementary table and search strategy.

## Figures and Tables

**Table 1:** Basic characteristics of the included studies.

Study ID	Cases (T/C)	Age (y)	Gender (M/F)	Physical status (KPS)	Stage	Experimental group		Control group (chemotherapy regimen)	Indicators
						Intervention	Number of KAI cycles		
Huang YN, 2011	286 (144/142)	42-71	194/92	KPS≥60	IIIa-IV	NP+ KAI(60ml/d, d1-d14)	2	NP	1,2,3,4,5,7
He JF, 2011	61 (31/30)	51-76	47/14	KPS>60	IIIa-IV	TP + KAI(40ml/d, d1-d14)	2	TP	1,2
Wei HY, 2013	64 (32/32)	37-65	42/22	KPS≥70	IIIb-IV	DP+ KAI(40ml/d, d1-d21)	2	DP	1,2,3,5,6,7,8
Sun LJ, 2008	108 (60/48)	45-74	86/22	KPS>70	III-IV	TP + KAI(50ml/d, d1-d30)	1	TP	1,2,3
Zou Y, 2013	76 (38/38)	52-74	52/24	KPS≥60	III-IV	NP+ KAI(40ml/d, d1-d21)	2	NP	1,2,4,5
Luo JH, 2009	64 (32/32)	30-75	51/13	KPS>60	IIIb-IV	NP+ KAI(40ml/d, d1-d21)	2	NP	1,2,3,4,5,8
Ma XP, 2012	52 (26/26)	31-76	33/19	KPS>60	IIIb-IV	DP+ KAI(30ml/d, d1-d30)	1	DP	1,2,3,4,5
Zhang XL, 2005	62 (30/32)	35-76	43/19	KPS>60	III-IV	NP+ KAI(40~60ml/d, d1-d20)	1	NP	1,2,3,8
Huang RW, 2006	50 (25/25)	35-78	34/16	KPS≥60	IIIb-IV	NP+ KAI(20~30ml/d, d1-d21)	2	NP	1,2,3
Jing H, 2007	97 (51/46)	35-73	61/36	KPS≥60	IIIa-IV	NP/VP + KAI(40ml/d, d1-d14)	3	NP/VP	1,2,4,5,7
Zou H, 2008	51 (26/25)	31-77	39/12	KPS≥60	IIIb-IV	DP + KAI(40~50ml/d, d1-d14)	2	DP	1,2,3
Wu DH, 2009	56 (28/28)	NR	43/13	KPS>60	IIIb-IV	TP + KAI(50ml/d, d1-d14)	2	TP	1,2,3,4,5
Wang LF, 2010	64 (32/32)	30-76	46/18	KPS≥70	III-IV	TP + KAI(50ml/d, d1-d7)	4	TP	1,2,3
Ge CZ, 2011	64 (32/32)	70-79	47/17	KPS≥60	IIIa-IV	GP+ KAI(30ml/d, d1-d21)	2	GP	1,2,3
Jiang L, 2011	65 (35/30)	50-79	44/21	KPS≥60	IIIb-IV	DP + KAI(40ml/d, d1-d12)	1	DP	1,2,3
Zheng ZP, 2012	48 (25/23)	NR	34/14	KPS>60	IIIb-IV	NP/TP/GP/PP + KAI(50ml/d, d1-d15)	4	NP/TP/GP/PP	3,8
Tu JG, 2012	63 (32/31)	70-85	41/22	KPS>60	IIIa-IV	DP + KAI(30ml/d, d1-d14)	2	DP	1,2,3,4,5,6,7
Chen L, 2014	83 (41/42)	NR	53/30	KPS>60	IIIa-IV	NP + KAI(40ml/d, d1-d21)	2	NP	1,2,8
Zhou ZY, 2014	80 (40/40)	62-76	44/36	KPS≥60	IIIb-IV	GP + KAI(40ml/d, d1-d14)	4	GP	1,2,8
Liu S, 2014	72 (36/36)	27-70	50/22	KPS≥70	IIIb-IV	TP/DP/GP/NP + KAI(60ml/d, d1-d14)	2	TP/DP/GP/NP	1,2,3,4,5,6,7
Huang JT, 2014	56 (28/28)	67-75	33/23	KPS>60	III-IV	TP + KAI(50ml/d, d1-d14)	2	TP	1,2,3,4,5,6,7
Wang ZF, 2017	76 (38/38)	21-74	54/22	KPS≥60	IV	FP + KAI(40ml/d, d1-d21)	1	FP	8
Luo JH, 2017	54 (27/27)	37-72	28/26	KPS≥60	IIIb-IV	NP + KAI(40ml/d, d1-d21)	2	NP	1,2
Yang XY, 2010	60 (30/30)	65-70	37/23	KPS>60	IIIb-IV	GP + KAI(40ml/d, d1-d14)	4	GP	1,2,3,8
Li JS, 2011	60 (30/30)	50-79	43/17	KPS≥60	IIIa-IV	TP + KAI(40ml/d, d1-d14)	3	TP	1,2,3,8
Wei HD, 2012	100 (50/50)	44-72	58/42	KPS≥60	IIIb-IV	NP + KAI(40ml/d, d1-d21)	4	NP	1,2,3,4,5
Li ZJ, 2013	68 (35/33)	43-75	45/23	KPS≥60	IIIa-IV	NP + KAI(40ml/d, d1-d10)	3	NP	1,2,3,5
Shi L, 2011	58 (29/29)	34-70	36/22	KPS>70	IIIb-IV	TP + KAI(30ml/d, d1-d14)	2	TP	1,2,3,4,5
Zhang MJ, 2009	120 (60/60)	29-75	76/44	KPS>60	IIIa-IV	TP + KAI(40ml/d, d1-d21)	2	TP	1,3
Wen JY, 2006	78 (40/38)	24-76	67/11	KPS>60	IIIb-IV	NP + KAI(50ml/d, d1-d14)	4	NP	1,2,3
Zhang JL, 2010	60 (30/30)	51-78	45/15	KPS>60	IIIa-IV	TP + KAI(40ml/d, d1-d14)	2	TP	1,2,3
Zhao JP, 2009	75 (38/37)	46-70	47/28	KPS≥60	IIIb-IV	DP + KAI(60ml/d, d1-d15)	2	DP	1,2,3,4,5
Hu DX, 2007	43 (21/22)	NR	29/14	KPS>60	IIIa-IV	NP + KAI(40~60ml/d, d1-d21)	2	NP	1,2
Guang XH, 2015	89 (46/43)	40-70	50/39	KPS≥60	IIIb-IV	GP + KAI(40ml/d, d1-d14)	2	GP	1,2,3,5
Zhang SQ, 2014	55 (30/25)	45-84	43/12	KPS≥60	III-IV	TP + KAI(60ml/d, d1-d21)	2	TP	1,2,3

KAI, Kangai injection; T, treatment; C, control; M, male; F, female; KPS, Karnofsky Performance Score; NR, not reported; GP, gemcitabine + platinum; TP, taxol + platinum; NP, navelbine + platinum; DP, docetaxel + platinum; PP, pemetrexed + platinum; FP, 5-Fluorouracil + platinum; VP, vindesine + platinum; ①Objective tumor response; ②Disease control rate; ③KPS/QOL(quality of life); ④gastrointestinal reaction (vomiting toxicity); ⑤leukocytopenia; ⑥hemoglobin deficiency; ⑦thrombocytopenia; ⑧immune function

**Table 2:** Sensitivity analysis of this study.

Immune index	*N*	MD (95%CI)	*I^2^*	Excluded articles^*^	*N*	MD (95%CI)	*I^2^*
CD_4_^+^	5	5.48 [2.68, 8.28]	86%	45, 48	3	8.01 [6.28, 9.73]	0%
CD_8_^+^	5	-2.37 [-4.51, -0.23]	81%	36,45,48	2	-6.27 [-8.86, -3.69]	0%
CD_4_^+^/CD_8_^+^	9	0.12 [-0.07, 0.30]	90%	19,45,48	6	0.26 [0.13, 0.38]	41%

*N*, the number of studies. ^*^reference number.

## Abbreviations

KAI: kangai injections
NSCLC: non-small cell lung cancer
RCT: randomized controlled trials
CMA: comprehensive meta-analysis
ORR: objective response rate
QOL: quality of life
TCM: traditional Chinese medicine
CPM: Chinese patent medicine
CNKI: Chinese National Knowledge Infrastructure
CBM: Chinese Biological Medicine
VIP: VIP Database for Chinese Technical Periodicals
PRISMA: Preferred Reported Items for Systematic Review and Meta-analysis
PROSPERO: International Prospective Register of Systematic Reviews
KPS: Karnofsky performance scores
WHO: World Health Organization
RECIST: Response Evaluation Criteria in Solid Tumors
CR: complete relief
PR: partial remission
SD: stable disease
PD: progressive disease
CMA: Comprehensive Meta-Analysis
RR: Risk ratio
MD: mean differences
CI: confidence intervals
TSA: Trial sequential analysis
MRA: meta-regression analysis
NK: natural killer cells
