# Supplementary material for: Kangai Injection Combined with Platinum-based Chemotherapy for the Treatment of Stage III/IV Non-Small Cell Lung Cancer: A Meta-analysis and Systematic Review of 35 Randomized Controlled Trials
Source: J Cancer. 2019 Aug 28;10(21):5283–98. doi: 10.7150/jca.31928 (PMC6775612; doi:10.7150/jca.31928)
Supplement: Supplementary file 1 — Supplementary table and search strategy. [file jcav10p5283s1.pdf]

Table S1: Methodological quality of the included studies evaluated using the Cochrane “risk of bias” tool.

References	Random sequence gengeration	Allocation concealment	Blinding of participants and personnel	Blinding of outcome assessment	Incomplete outcome data	Selective reporting	other bias
Huang YN, 2011	?	?	?	?	?	+	?
He JF, 2011	+	?	?	?	?	–	?
Wei HY, 2013	+	?	?	?	+	+	?
Sun LJ, 2008	?	?	?	?	?	+	?
Zou Y, 2013	?	?	?	?	+	+	?
Luo JH, 2009	?	?	?	?	?	?	?
Ma XP, 2012	?	?	?	?	?	+	?
Zhang XL, 2005	?	?	?	?	?	+	?
Huang RW, 2006	?	?	?	?	?	+	?
Jing H, 2007	+	?	?	?	+	+	?
Zou H, 2008	?	?	?	?	?	+	?
Wu DH, 2009	+	?	?	?	?	+	?
Wang LF, 2010	?	?	?	?	?	+	?
Ge CZ, 2011	?	?	?	?	?	–	?
Jiang L, 2011	?	?	?	?	?	+	?
Zheng ZP, 2012	+	+	?	?	?	+	?
Tu JG, 2012	?	?	?	?	?	+	?
Chen L, 2014	+	?	?	?	?	+	?
Zhou ZY, 2014	?	?	?	?	?	+	?
Liu S, 2014	?	?	?	?	?	+	?
Huang JT, 2014	?	?	?	?	?	+	?
Wang ZF, 2017	?	?	?	?	?	+	?
Luo JH, 2017	?	?	?	?	?	–	?
Yang XY, 2010	?	?	?	?	+	+	?

Li JS, 2011	+	?	?	?	?	+	?
Wei HD, 2012	?	?	?	?	+	+	?
Li ZJ, 2013	?	?	?	?	?	+	?
Shi L, 2011	?	?	?	?	?	+	?
Zhang MJ, 2009	?	?	?	?	?	+	?
Wen JY, 2006	?	?	?	?	?	+	?
Zhang JL, 2010	+	?	?	?	?	+	?
Zhao JP, 2009	?	?	?	?	?	+	?
Hu DX, 2007	?	?	?	?	+	+	?
Guang XH, 2015	+	?	?	?	?	+	?
Zhang SQ, 2014	?	?	?	?	?	−	?

+ = low risk of bias; ? = unclear risk of bias; - = high risk of bias

Supplementary materials

Search strategy

PubMed

#1 (((non small cell lung cancer[Title/Abstract]) OR non-small cell lung cancer[Title/Abstract]) OR NSCLC[Title/Abstract]) OR (Lung cancer[Mesh] OR Non-small Cell Lung Cancer[Mesh])

#2 ((Kangai[Title/Abstract]) OR Kang ai[Title/Abstract]) OR KAI[Title/Abstract]

#3 ("RCT*" OR "randomized controlled trial" OR "Randomized Controlled Trial" [Publication Type] OR "Randomized Controlled Trials as Topic"[Mesh] OR "Controlled Clinical Trial" [Publication Type])

#4 #1 and #2 and #3
